# Biochemical Characterization and Active-Site Analysis of *N*-Acetylornithine Aminotransferase from *Crocosphaera subtropica* ATCC 51142

**DOI:** 10.3390/life16071212

**Published:** 2026-07-22

**Authors:** Liyang Huang, Zhi-Min Li, Luna Gao, Siqi Wang, Zhifeng Wu, Zhimin Li

**Affiliations:** 1 Jiangxi Engineering Laboratory for the Development and Utilization of Agricultural Microbial Resources, College of Bioscience and Bioengineering, Jiangxi Agricultural University, Nanchang 330045, Chinawangsiqi3482@126.com (S.W.);; 2 College of Chemistry and Materials, Jiangxi Agricultural University, Nanchang 330045, China

**Keywords:** *Crocosphaera subtropica* ATCC 51142, *N*-acetylornithine aminotransferase, molecular docking, molecular dynamics simulations, site-directed mutagenesis

## Abstract

*N*-acetylornithine aminotransferase (AcOAT) is a pyridoxal 5′-phosphate (PLP)-dependent enzyme that catalyzes a key transamination step in arginine biosynthesis. In cyanobacteria, arginine metabolism is closely associated with nitrogen assimilation and storage, yet biochemical information on cyanobacterial AcOATs remains limited. In this study, the AcOAT encoded by the *cce_3094* gene from *Crocosphaera subtropica* ATCC 51142 (*Cs*AcOAT) was cloned, heterologously expressed, purified, and systematically characterized. Recombinant *Cs*AcOAT was obtained as a soluble protein with an apparent molecular mass of approximately 43 kDa. Steady-state kinetic analysis showed that *Cs*AcOAT catalyzed transamination between *N*-acetylornithine (AcOrn) and α-ketoglutarate (α-KG), with apparent *K*_M_ values of 0.17 ± 0.03 mM for AcOrn and 0.020 ± 0.003 mM for α-KG, indicating a higher affinity for α-KG. The enzyme exhibited optimal activity at pH 8.5 and 30 °C, retained relatively high activity over a broad temperature range of 0–50 °C, and was activated by Zn^2+^ and Co^2+^ but inhibited by Ni^2+^. Structural analysis based on homology modeling, molecular docking, and molecular dynamics simulations suggested a conserved PLP-dependent aminotransferase fold and a stable binding mode for the PLP-AcOrn complex in the active-site pocket. Site-directed mutagenesis further demonstrated that Gly114, Asp239, Lys268, and Thr296 are indispensable for catalytic activity, whereas Ser113, Ala115, and Gln242 make important contributions to catalytic turnover and cofactor-assisted catalysis. These results provide biochemical and structural characterization of *Cs*AcOAT, expand current knowledge of cyanobacterial AcOATs, and offer a useful basis for future studies on arginine metabolism and nitrogen storage in diazotrophic cyanobacteria.

## 1. Introduction

Cyanobacteria were among the earliest oxygenic phototrophs on Earth and make major contributions to global carbon and nitrogen cycling [1,2]. *Crocosphaera subtropica* ATCC 51142 (formerly *Cyanothece* sp. ATCC 51142) is a model unicellular marine diazotrophic cyanobacterium because it performs both oxygenic photosynthesis and aerobic nitrogen fixation within a single cell [3]. These two incompatible processes are separated in time: photosynthesis occurs during the light period, whereas nitrogen fixation is restricted to the dark period [4]. In this organism, cyanophycin serves as a transient nitrogen reservoir. It accumulates during dark-phase nitrogen fixation and is later mobilized to support metabolism during the light phase [5]. Arginine biosynthesis is therefore relevant not only to protein synthesis, but also to cyanophycin metabolism and, more broadly, to the coordination of nitrogen assimilation with central carbon metabolism in *C. subtropica* ATCC 51142 [6,7].

**Figure 1:**
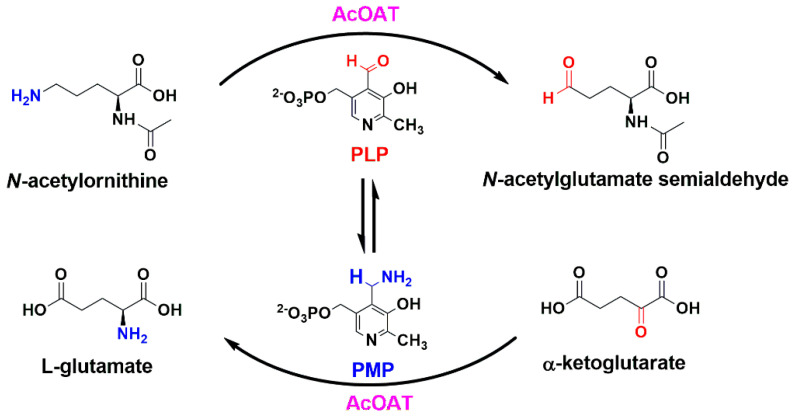
The reaction catalyzed by AcOAT.

*N*-acetylornithine aminotransferase (AcOAT, EC 2.6.1.11) is a pyridoxal 5′-phosphate (PLP)-dependent enzyme that catalyzes the reversible transamination between *N*-acetylornithine (AcOrn) and α-ketoglutarate (α-KG), generating *N*-acetylglutamate semialdehyde and L-glutamate (Figure 1) [8]. This reaction links the ornithine branch of arginine biosynthesis to the α-KG node of central metabolism [9]. In *C. subtropica* ATCC 51142, AcOAT is encoded by the *cce_3094* (*argD*) gene [10]. Given the role of arginine as a precursor for cyanophycin, the properties of this enzyme may be relevant to nitrogen storage and redistribution in this strain.

AcOATs from several heterotrophic bacteria, including *Escherichia coli*, *Salmonella typhimurium*, *Mycobacterium tuberculosis*, and *Corynebacterium glutamicum*, have been characterized at the biochemical and structural levels [11,12,13,14]. These studies showed that the catalytic framework of AcOAT is broadly conserved, whereas substrate preference and enzymatic behavior can vary across species. For instance, the *E. coli* enzyme accepts both AcOrn and *N*-succinyl-L,L-diaminopimelate, linking it to both arginine and lysine biosynthesis [12]. By contrast, AcOAT from *S. typhimurium* shows higher specificity for AcOrn than the homolog from *Thermus thermophilus* HB8, apparently because of subtle differences in active-site architecture [13]. AcOAT from *Thermotoga maritima* is notable for its high-temperature activity and has been considered a useful scaffold for industrial enzyme engineering [15]. Despite these advances, cyanobacterial AcOATs remain much less explored. Biochemical characterization has so far been limited mainly to enzymes from *Synechococcus* sp. PCC 7002 and *Synechocystis* sp. PCC 6803 [16,17]. Additional studies from phylogenetically and physiologically distinct cyanobacteria are needed to better understand the diversity of this enzyme family.

In the present study, we cloned, expressed, and characterized the AcOAT from *C. subtropica* ATCC 51142 (*Cs*AcOAT). We examined its kinetic properties, pH and temperature dependence, and responses to metal ions. We also used homology modeling, molecular docking, molecular dynamics (MD) simulations, and site-directed mutagenesis to investigate the catalytic pocket and test the roles of conserved residues. This work extends current knowledge of cyanobacterial AcOATs and provides a basis for future studies of arginine metabolism in diazotrophic cyanobacteria.

## 2. Materials and Methods

### 2.1. Materials

*Escherichia coli* DH5α and BL21(DE3) strains and the pET-28a expression vector were available in our laboratory. Genomic DNA of *C. subtropica* ATCC 51142 was purchased from the American Type Culture Collection (Manassas, VA, USA). Oligonucleotides were synthesized, and DNA sequencing was performed by GENEWIZ (Hangzhou, China). The site-directed mutagenesis kit, restriction endonucleases, DNA polymerase, T4 DNA ligase, DNA and protein molecular weight markers, and Ni-NTA resin were purchased from TransGen Bio-tech (Beijing, China). Unless otherwise stated, all other chemicals were of analytical grade and obtained from Solarbio Life Sciences (Beijing, China).

### 2.2. Cloning of the cce_3094 Gene

The *cce_3094* gene (GenBank: ACB52442.1) was amplified by polymerase chain reaction (PCR) using genomic DNA of *C. subtropica* ATCC 51142 as template and the primers listed in Table S1. The PCR mixture contained 30 ng template DNA, 0.2 μM forward and reverse primers (2 μL each), 25 μL 2 × Pfu PCR Master Mix, and nuclease-free water to a final volume of 50 μL. PCR was performed under the following conditions: initial denaturation at 95 °C for 5 min; 30 cycles of 95 °C for 30 s, 56 °C for 30 s, and 72 °C for 2 min; and a final extension at 72 °C for 10 min. The amplified fragment was inserted into the pET-28a vector by restriction digestion and ligation to generate the recombinant plasmid pET28a-cce3094, which was verified by DNA sequencing.

### 2.3. Expression and Purification of Recombinant CsAcOAT

A single *E. coli* BL21(DE3) colony harboring pET28a-cce3094 was inoculated into LB medium supplemented with kanamycin (50 μg/mL) and cultured overnight at 37 °C with shaking at 180 rpm. The overnight culture was transferred at 1% (*v*/*v*) into fresh LB medium containing kanamycin and grown under the same conditions until the OD_600_ reached approximately 0.6. After incubation on ice for 30 min, protein expression was induced with 0.2 mM isopropyl-β-D-thiogalactopyranoside (IPTG), and the culture was further incubated at 16 °C for 24 h with shaking at 180 rpm. Cells were harvested by centrifugation at 8000× *g* for 10 min at 4 °C, washed twice with lysis buffer (20 mM Tris-HCl, pH 7.5), and resuspended in 20 volumes of the same buffer. The cells were disrupted by sonication on ice, and cell debris was removed by centrifugation at 10,000× *g* for 60 min at 4 °C. The supernatant was loaded onto a Ni-NTA affinity column, and the recombinant protein was eluted with lysis buffer containing 20–200 mM imidazole. The purified protein was dialyzed against storage buffer consisting of 20 mM Tris-HCl (pH 7.5), 100 mM NaCl, and 10% glycerol, and then concentrated for subsequent experiments. Protein purity was analyzed by 12% SDS-PAGE.

### 2.4. Site-Directed Mutagenesis

Site-directed mutagenesis was carried out using a commercial mutagenesis kit with pET28a-cce3094 as the template and the primers listed in Table S1. All mutant proteins were expressed and purified using the same procedure as for the wild-type protein.

### 2.5. Kinetic Characterization of CsAcOAT

The activity of *Cs*AcOAT was determined using a glutamate dehydrogenase (GDH)-coupled assay as described previously [13]. The standard reaction mixture (200 μL) contained 100 mM Tris-HCl (pH 8.2), 0.1 mM PLP, 2 mM NADP^+^, 4 U GDH, and 0.2 μM *Cs*AcOAT. To determine the kinetic parameters for *N*-acetylornithine (AcOrn), the concentration of AcOrn was varied from 0.05 to 2 mM, whereas α-KG was maintained at a fixed saturating concentration of 0.2 mM. To determine the kinetic parameters for α-KG, the concentration of α-KG was varied from 0.005 to 0.2 mM, whereas AcOrn was maintained at a fixed saturating concentration of 2 mM. After equilibration at 25 °C for 5 min, the reaction was initiated by addition of the enzyme. NADPH formation was monitored at 340 nm using a microplate reader, and absorbance was recorded every 5 s for 10 min. Initial velocities were calculated from the linear increase in absorbance using the extinction coefficient of NADPH (ε = 6220 M^−1^·cm^−1^). Kinetic parameters were obtained by nonlinear regression fitting to the Michaelis–Menten equation using KaleidaGraph 4.0 (Synergy Software). All measurements were performed in triplicate using independent enzyme preparations.

### 2.6. Determination of the Effects of pH, Temperature, and Metal Ions on CsAcOAT Activity

The effect of pH on *Cs*AcOAT activity was determined using a two-step discontinuous assay. The primary reaction mixture (500 μL) contained 2 mM AcOrn, 0.2 mM α-KG, 0.1 mM PLP, and 0.2 μM *Cs*AcOAT in buffers of different pH values (pH 6.0–9.0, Table S2), and was incubated at 25 °C for 10 min. The reaction was terminated by heating at 95 °C for 5 min, followed by centrifugation at 12,000× *g* for 10 min at 4 °C to remove precipitated protein. An aliquot (40 μL) of the resulting supernatant was added to a secondary reaction mixture containing 2 mM NADP^+^ and 4 U GDH in 100 mM Tris-HCl buffer (pH 8.2) to a final volume of 200 μL. NADPH formation was then measured at 340 nm as described above. Relative activity was calculated based on the amount of NADPH produced under each pH condition. The effects of temperature and metal ions on *Cs*AcOAT activity were determined using the same assay system under the indicated conditions [16]. For temperature profiling, the primary reaction was performed at 0–60 °C. For metal ion analysis, enzyme activity was measured in the presence of 2 mM of the indicated metal ions (Na^+^, Mg^2+^, Cu^2+^, Mn^2+^, Zn^2+^, Ca^2+^, Co^2+^, and Ni^2+^).

### 2.7. Bioinformatics Analysis and Homology Modeling

The amino acid sequence of *Cs*AcOAT was deduced from the nucleotide sequence deposited in GenBank (accession no. ACB52442.1). Its physicochemical properties, including molecular weight, theoretical isoelectric point, average hydrophilicity, and extinction coefficient, were analyzed using ProtParam (https://web.expasy.org/protparam/, accessed on 25 September 2025). Secondary structure was predicted using the SOPMA online server (https://npsa-prabi.ibcp.fr/, accessed on 17 March 2026). Multiple sequence alignment of *Cs*AcOAT with structurally characterized AcOAT homologs was performed using Clustal Omega [18]. The sequence alignment was visualized using ESPript 3.0 [19]. A homology model of *Cs*AcOAT was generated with SWISS-MODEL [20] using the crystal structure of AcOAT from *Thermotoga maritima* (PDB ID: 2ORD) as the template, which shared 47.14% sequence identity with *Cs*AcOAT. In addition, AlphaFold and RoseTTAFold were used to predict the three-dimensional structure of *Cs*AcOAT for comparison and validation of the modeled structure [21,22].

### 2.8. Molecular Docking Analysis

Molecular docking was performed using AutoDock Tools 1.5.7 [23] to investigate the interactions of *Cs*AcOAT with PLP and AcOrn. The homology model of *Cs*AcOAT was used as the receptor. Ligand structures were obtained from related crystal structures (PDB IDs: 1WKG and 2E54) and prepared by adding Gasteiger charges and assigning rotatable bonds. The receptor protein was prepared by adding hydrogen atoms and assigning atomic charges. Docking simulations were carried out using the Lamarckian genetic algorithm under a semi-flexible docking protocol, with the grid box centered on the predicted active site. The docking poses with the lowest predicted binding energies were selected for further analysis. Protein–ligand interactions, including hydrogen-bonding, hydrophobic, and electrostatic interactions, were analyzed by Protein Ligand Interaction Profiler (PLIP) [24] and visualized using PyMOL.

### 2.9. Molecular Dynamics Simulations

Molecular dynamics (MD) simulations were performed using GROMACS 2022.2 [25]. The protein was described with the Amber14SB force field [26], and ligand parameters were generated using GAFF2 with AM1-BCC atomic charges [27,28]. Ligand topologies were converted into GROMACS-compatible formats using ACPYPE [29]. The complex of *Cs*AcOAT docked with PLP-AcOrn was placed in a truncated dodecahedral box with a minimum distance of 1.2 nm from the box edge, solvated with TIP3P water [30], and neutralized with Na^+^/Cl^−^ ions to 0.15 M using Joung–Cheatham parameters [31]. Energy minimization was performed using the steepest descent algorithm until the maximum force was below 1000 kJ·mol^−1^·nm^−1^, followed by 200 ps NVT and 200 ps NPT equilibration at 298 K using the V-rescale thermostat and Berendsen barostat. A 100 ns production simulation was then carried out in the NPT ensemble with a 2 fs integration time step using the Nose–Hoover thermostat and Parrinello–Rahman barostat to maintain 298 K and 1 bar, respectively [32]. Long-range electrostatic interactions were treated using particle mesh Ewald (PME) method with a 1.2 nm cutoff for Coulombic and van der Waals interactions [33]. All bonds involving hydrogen atoms were constrained using the LINCS algorithm [34]. Trajectory coordinates were saved every 10 ps. Structural analyses were conducted using GROMACS tools, VMD, and PyMOL. Binding free energies were estimated using the MM-PBSA method implemented in gmx_MMPBSA [35].

## 3. Results and Discussion

### 3.1. Bioinformatics Analysis of CsAcOAT

The *cce_3094* gene is 1269 bp long and encodes *Cs*AcOAT of 422 amino acids [10]. ProtParam analysis predicted a molecular weight of 46,303.03 Da and a theoretical isoelectric point (pI) of 5.24, indicating that *Cs*AcOAT is an acidic protein. The extinction coefficient was estimated to be 38,890 M^−1^·cm^−1^. The aliphatic index and grand average of hydropathicity (GRAVY) value were 94.81 and −0.061, respectively, suggesting that the protein is relatively thermostable and overall hydrophilic [36,37].

Secondary structure prediction showed that *Cs*AcOAT is dominated by α-helices (43.60%), followed by random coils (30.33%), extended strands (16.82%), and β-turns (9.24%). This overall pattern is compatible with the fold commonly observed in PLP-dependent aminotransferases. At this stage, these sequence-based features mainly provide a general description of the protein rather than a functional explanation, but they are consistent with subsequent structural modeling.

### 3.2. Cloning, Expression and Purification of CsAcOAT

**Figure 2:**
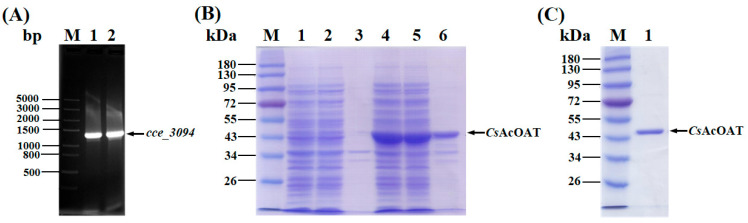
Cloning, expression, and purification of recombinant wild-type *Cs*AcOAT. (**A**) PCR amplification of the *cce_3094* gene. M: DNA marker, Lanes 1–2: PCR product. (**B**) SDS-PAGE analysis of recombinant *Cs*AcOAT expression. M: protein marker, Lanes 1–3: whole-cell lysate, supernatant, and pellet fraction of *E. coli* BL21(DE3) harboring pET28-a plasmid, respectively, Lanes 4–6: whole-cell lysate, supernatant, and pellet fraction of *E. coli* BL21(DE3) harboring plasmid pET28a-cce3094, respectively. (**C**) SDS-PAGE analysis of purified *Cs*AcOAT. M: protein marker, Lane 1: purified *Cs*AcOAT.

The *cce_3094* gene encoding *Cs*AcOAT was amplified via high-fidelity PCR, giving a distinct DNA fragment of approximately 1300 bp, as expected for the 1269 bp gene (Figure 2A). After sequence verification, the recombinant plasmid pET28a-cce3094 was transformed into *E. coli* BL21(DE3) competent cells for protein expression. SDS-PAGE showed that the recombinant *Cs*AcOAT was expressed predominantly in soluble form, with an apparent molecular mass of about 43 kDa, close to the calculated value of 46 kDa (Figure 2B). The protein was purified by Ni-NTA affinity chromatography and eluted efficiently with lysis buffer containing 60 mM imidazole, yielding a highly purified preparation (Figure 2C and Figure S1). These findings were consistent with the bioinformatically predicted characteristics of *Cs*AcOAT. The final protein concentration reached 205 μM, corresponding to a yield of approximately 9.84 mg/g wet cell mass. These results established a workable system for biochemical characterization of *Cs*AcOAT.

### 3.3. Steady-State Kinetic Characterization of CsAcOAT

**Figure 3:**
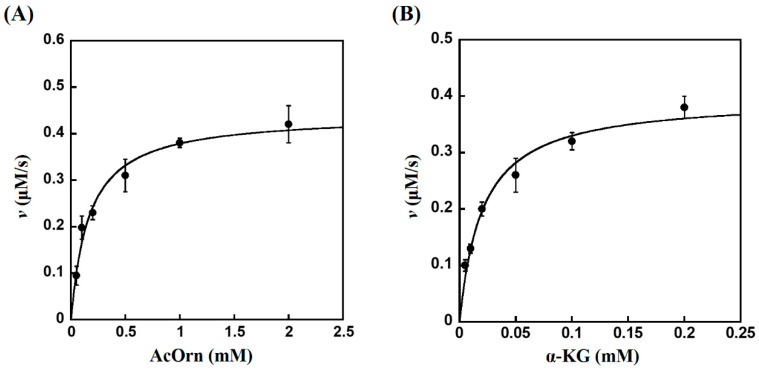
Steady-state kinetic characterization of *Cs*AcOAT. Enzyme assays were performed in a 200 μL reaction mixture containing 0.05–2 mM AcOrn at a fixed α-KG concentration of 0.2 mM (**A**), or 0.005–0.2 mM α-KG at a fixed AcOrn concentration of 2 mM (**B**), together with 0.1 mM PLP, 2 mM NAD^+^, 4 U GDH, and 0.2 μM *Cs*AcOAT in 100 mM Tris-HCl buffer (pH 8.2). Each measurement was performed in triplicate using independent enzyme preparations.

The steady-state kinetics of *Cs*AcOAT were determined using AcOrn and α-KG as variable substrates in the presence of PLP (Figure 3). When the concentration of α-KG was fixed at 0.2 mM, the apparent maximum initial velocity (*V*_max_) was 0.44 ± 0.02 μM/s, and the Michaelis constant (*K_M_*) value of AcOrn was 0.17 ± 0.03 mM (Figure 3A). Based on an enzyme concentration of 0.2 μM, the corresponding catalytic rate (*k*_cat_) and catalytic efficiency (*k*_cat_/*K_M_*) were calculated to be 2.2 ± 0.1 s^−1^ and 1.29 × 10^4^ M^−1^·s^−1^, respectively. When AcOrn was fixed at 2 mM, the apparent *V*_max_ for α-KG was 0.40 ± 0.03 μM/s, with a *K_M_* value of 0.020 ± 0.003 mM (Figure 3B). The resulting *k*_cat_ and *k*_cat_/*K_M_* values were 2.0 ± 0.2 s^−1^ and 1.0 × 10^5^ M^−1^·s^−1^, respectively. Thus, *Cs*AcOAT showed an approximately 8.5-fold lower *K_M_* and 7.7-fold higher *k*_cat_/*K_M_* for α-KG than for AcOrn, indicating a clear preference for α-KG in this two-substrate system.

The *K_M_* value of *Cs*AcOAT for AcOrn (0.17 mM) is close to those reported for the enzymes from *E. coli* (0.15 mM) and *Synechocystis* sp. PCC 6803 (0.12 mM) [12,16], but is approximately 2.5-fold higher than that of the enzyme from *Corynebacterium glutamicum* [11]. Its catalytic efficiency toward AcOrn is also higher than the value reported for the *E. coli* enzyme (4.0 × 10^3^ M^−1^·s^−1^) [12]. In contrast, AcOAT from *Salmonella typhimurium* exhibited both higher substrate affinity and higher catalytic efficiency, with a *K_M_* of 0.037 mM and a *k*_cat_/*K_M_* of 4.2 × 10^5^ M^−1^·s^−1^ for AcOrn [13].

One feature of note is its relatively high affinity for α-KG. Because α-KG occupies a central position in carbon and nitrogen metabolism and is often maintained at modest intracellular levels [38], strong α-KG binding may help sustain arginine-pathway flux under changing metabolic conditions. Whether this property is specifically linked to nitrogen storage physiology in *C. subtropica* remains unresolved, but it is consistent with a role for *Cs*AcOAT at the interface of amino acid biosynthesis and central metabolism.

### 3.4. Effects of pH, Temperature, and Metal Ions on CsAcOAT Activity

**Figure 4:**
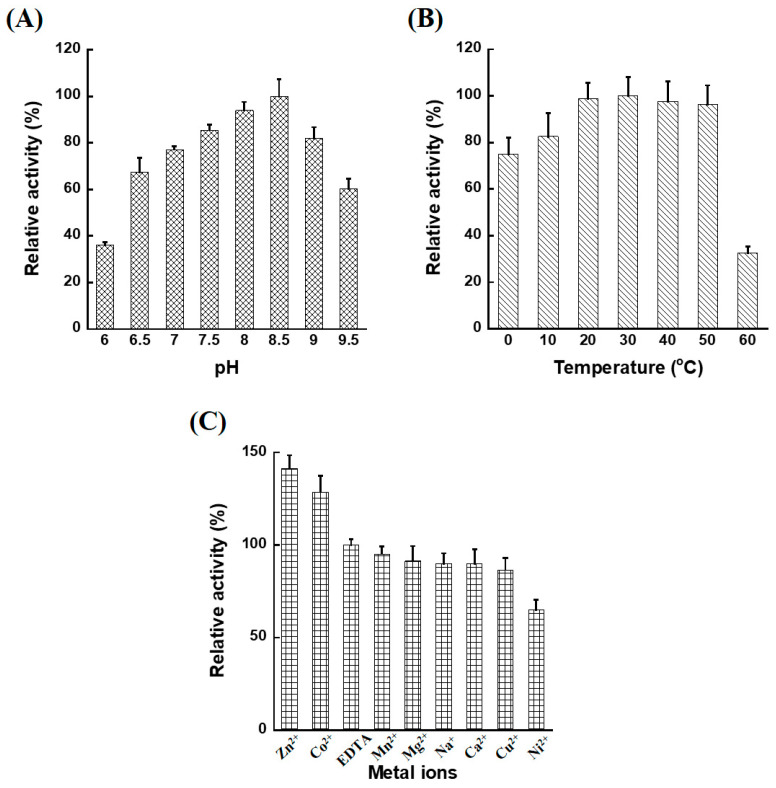
Effects of pH, temperature and metal ions on *Cs*AcOAT activity. (**A**) Effect of pH on *Cs*AcOAT activity. Relative activity was calculated with the activity at pH 8.5 defined as 100%. (**B**) Effect of temperature on *Cs*AcOAT activity. Relative activity was calculated with the activity at 30 °C defined as 100%. (**C**) Effect of different metal ions (2 mM each) on *Cs*AcOAT activity. Relative activity was calculated with the activity in the presence of 2 mM EDTA defined as 100%. Data are presented as the mean ± SD of three independent experiments.

The optimal pH for *Cs*AcOAT activity was 8.5 (Figure 4A). The enzyme retained relatively high activity over a broad pH range from 7.0 to 9.0, although its activity under alkaline conditions was markedly higher than that under acidic conditions. This pH preference is consistent with that reported for AcOATs from other organisms, which generally exhibit maximal activity in alkaline environments. For example, AcOAT from *Pseudomonas aeruginosa* has an optimal pH of 8.5 and retains high activity between pH 7.5 and 9.5 [39].

The optimal temperature for *Cs*AcOAT activity was 30 °C (Figure 4B). The enzyme maintained more than 80% of its maximal activity over a temperature range of 0–50 °C, indicating broad temperature adaptability. A similar pattern has been reported for other AcOATs. For instance, AcOAT from *Corynebacterium crenatum* exhibits an optimal temperature of 30 °C and retains about 90% of its maximal activity from 5 to 55 °C [40]. In contrast, AcOAT from *Synechocystis* sp. PCC6803 shows pronounced activity changes over the temperature range of 15–55 °C [16]. However, the activity of *Cs*AcOAT declined sharply at temperatures above 60 °C (Figure 4B), indicating that *Cs*AcOAT is not unusually thermotolerant despite its relatively high aliphatic index.

Metal-ion effects were assessed using 2 mM of each ion, with 2 mM EDTA as the reference condition (Figure 4C). Among the tested ions, Zn^2+^ and Co^2+^ enhanced activity by 41% and 28%, respectively, whereas Ni^2+^ reduced activity by about 30%. Mn^2+^, Mg^2+^, Na^+^, Ca^2+^, and Cu^2+^ caused little or no obvious change under the assay condition (Figure 4C). The basis for the species-dependent effects of metal ions on AcOAT activity remains unclear. For example, Zn^2+^ inhibits the activity of AcOAT from *Synechocystis* sp. PCC6803 by 90% [16], but acts as an activator for *Cs*AcOAT. Likewise, Mn^2+^ markedly activates the enzyme from *Synechocystis* sp. PCC 6803 [16], but has only a minor effect on *Cs*AcOAT and the *E. coli* enzyme [12].

### 3.5. Structure Modeling and Docking Analysis of CsAcOAT

**Figure 5:**
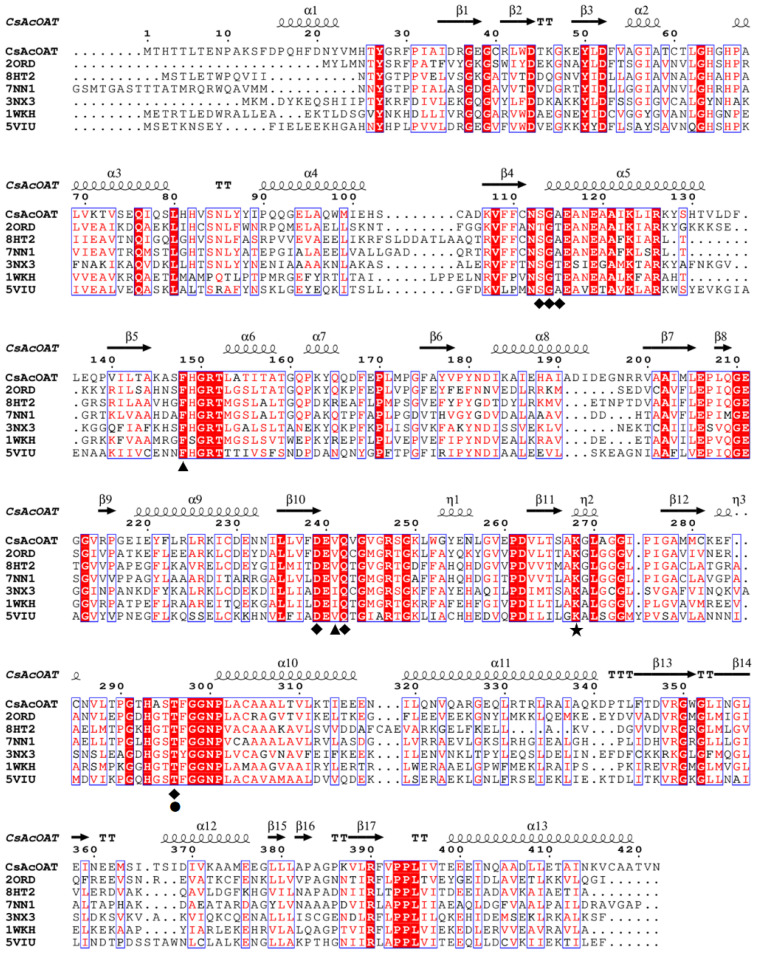
Multiple sequence alignment of *Cs*AcOAT with AcOAT homologs of known crystal structure from diverse organisms. *Cs*AcOAT: *Crocosphaera subtropica* ATCC 51142; 2ORD: *Thermotoga maritima*; 8HT2: *Corynebacterium glutamicum*; 7NN1: *Mycobacterium tuberculosis*; 3NX3: *Campylobacter jejuni*; 1WKH: *Thermus thermophilus* HB8; 5VIU: *Elizabethkingia anophelis*. Residues involved in hydrogen-bond interactions are indicated by solid diamonds (⬥), and hydrophobic interaction sites are marked by triangles (▲). The key residue involved in Schiff base formation is denoted by a star (★), and the inter-chain residue is indicated by a solid circle (●). Secondary structural elements of *Cs*AcOAT are shown above the aligned sequences, and residue numbering corresponds to that of *Cs*AcOAT.

To examine whether *Cs*AcOAT retains the characteristic features of AcOAT family proteins, we aligned its sequence with six homologs of known structure in PDB (Figure 5). *Cs*AcOAT shares relatively low overall sequence identity with previously crystallized AcOATs (Table S3), with the highest identity (47.14%) observed for the homolog from *Thermotoga maritima* (PDB ID: 2ORD). Although the overall sequence conservation is limited, residues involved in PLP binding and catalysis are highly conserved among the aligned sequences (Figure 5). In particular, Gly114, Asp239, Gln242, and Thr296 (from the other monomer), which are associated with hydrogen-bonding interactions with cofactor in other AcOATs [11], are retained in *Cs*AcOAT. Phe148, which contributes hydrophobic packing, and Lys268, the catalytic that forms the internal Schiff base with PLP, are also strictly conserved [13]. Thus, despite substantial divergence at the primary-sequence level, the catalytic core appears to be preserved.

**Figure 6:**
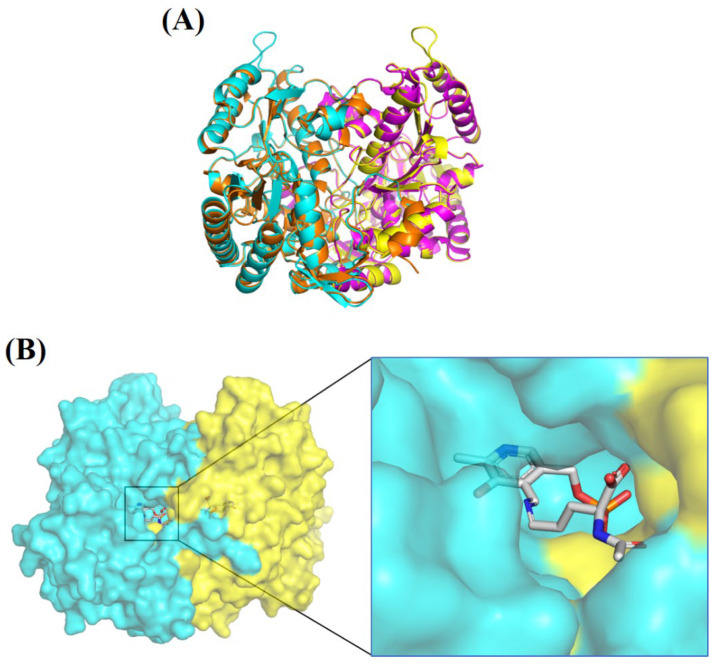
Structural model of *Cs*AcOAT generated using SWISS-MODEL. (**A**) Structural alignment of *Cs*AcOAT with *T. maritima* AcOAT (PDB ID: 2ORD). *Cs*AcOAT is shown in cyan and yellow, whereas *T. maritima* AcOAT is shown in orange and magenta. (**B**) Active-site pocket of *Cs*AcOAT. The pocket was identified by structural alignment of the *Cs*AcOAT model with AcOAT from *T. thermophilus* HB8 (PDB ID: 1WKG). The stick representation shows the PLP-AcOrn complex from 1WKG. The two subunits of *Cs*AcOAT are colored cyan and yellow, respectively.

A three-dimensional model of *Cs*AcOAT was then constructed using SWISS-MODEL with the crystal structure of *Thermotoga maritima* AcOAT (PDB ID: 2ORD) as the template (Figure 6). The homology model was acceptable, with a GMQE score of 0.76 and a QMEANDisCo global score of 0.78; more than 90% of residues had QMEANDisCo local scores above 0.6 (Figure S2). AlphaFold and RoseTTAFold predictions provided an independent point of comparison, and all three models were highly similar, with pairwise RMSD values of 0.677–0.907 Å (Figure S3). AlphaFold prediction likewise showed a strong confidence, with a pTM score of 0.95 and pLDDT values greater than 90 across most regions of the protein (Figure S4). Alignment of the SWISS-MODEL structure with the *T. maritima* AcOAT template gave an RMSD of 0.216 Å (Figure 6A), supporting conservation of the overall fold.

The modeled structure is consistent with the architecture expected for PLP-dependent aminotransferases such as γ-aminobutyrate aminotransferase from *Corynebacterium glutamicum* (PDB ID: 6J2V) and aspartate aminotransferase from *Thermus thermophilus* HB8 (PDB ID: 1GCK) (Figure S5) [41,42]. An apparent active-site pocket was identified in a region corresponding to the catalytic center of previously characterized AcOATs from *C. glutamicum* and *Mycobacterium tuberculosis* (Figure 6B) [11,14]. Similar pockets are also present in the AcOAT structures from *T. thermophilus* HB8 (PDB ID: 1WKG) and *T. maritima* (PDB ID: 2E54), pointing to a conserved organization of the catalytic region among these enzymes. This observation does not by itself establish the full catalytic mechanism, but it supports the idea that *Cs*AcOAT uses the same general active-site framework as other members of the family.

**Figure 7:**
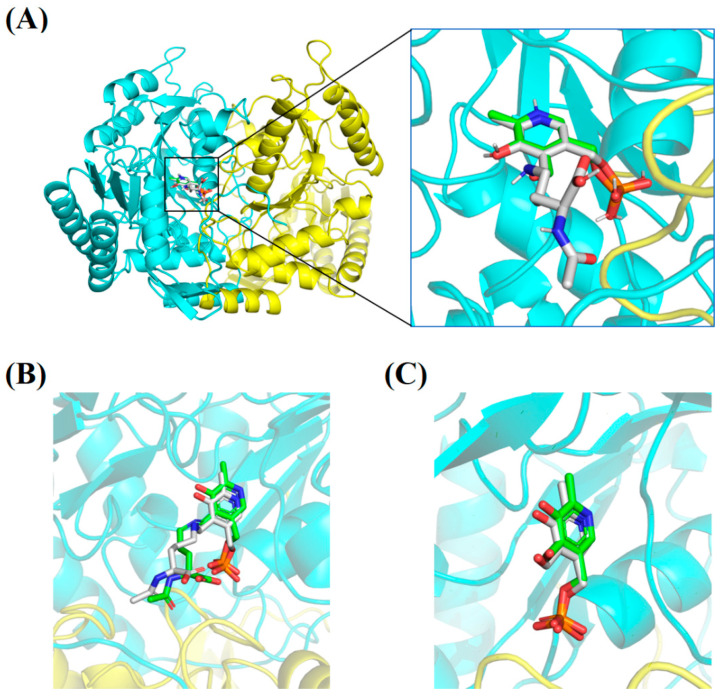
Molecular docking analysis of PLP or PLP-AcOrn in the predicted active site of *Cs*AcOAT. (**A**) Superposition of PLP and PLP–AcOrn after independent docking into the SWISS-MODEL-generated structure of *Cs*AcOAT. The PLP moiety of PLP–AcOrn occupies a position nearly identical to that of PLP. Carbon atoms of PLP-AcOrn and PLP are shown in gray and green, respectively. (**B**) Comparison of the docked PLP–AcOrn pose in *Cs*AcOAT with that in the crystal structure of AcOAT from *T. thermophilus* HB8 (PDB ID: 1WKG). Carbon atoms in the model and crystal structure are shown in gray and green, respectively. (**C**) Superposition of docked PLP in *Cs*AcOAT with PLP in the crystal structure of AcOAT from *T. maritima* (PDB ID: 2ORD). Carbon atoms in the model and crystal structure are shown in gray and green, respectively.

Molecular docking was then performed using the SWISS-MODEL-generated structure of *Cs*AcOAT with PLP or the PLP-AcOrn complex as the ligand. When docked separately, the PLP moiety of PLP-AcOrn occupied nearly the same position as PLP alone (Figure 7A). The lowest predicted binding energies were −7.9 kcal/mol for PLP and −8.1 kcal/mol for PLP-AcOrn (Table S4), indicating that both ligands exhibit strong binding affinity toward the protein [43,44]. Furthermore, the docked PLP-AcOrn complex adopted a binding pose highly similar to that observed in the crystal structure of AcOAT from *T. thermophilus* HB8 (PDB ID: 1WKG) (Figure 7B). Likewise, the position of the PLP cofactor in the docked model nearly overlapped with that in the crystal structure of AcOAT from *T. maritima* (PDB ID: 2ORD) (Figure 7C). These structural consistencies support the plausibility of modeled active site and provide a reasonable structural basis for subsequent mutational analysis, although docking alone cannot define the entire catalytic sequence.

### 3.6. MD Simulations Support Stable Ligand Binding

**Figure 8:**
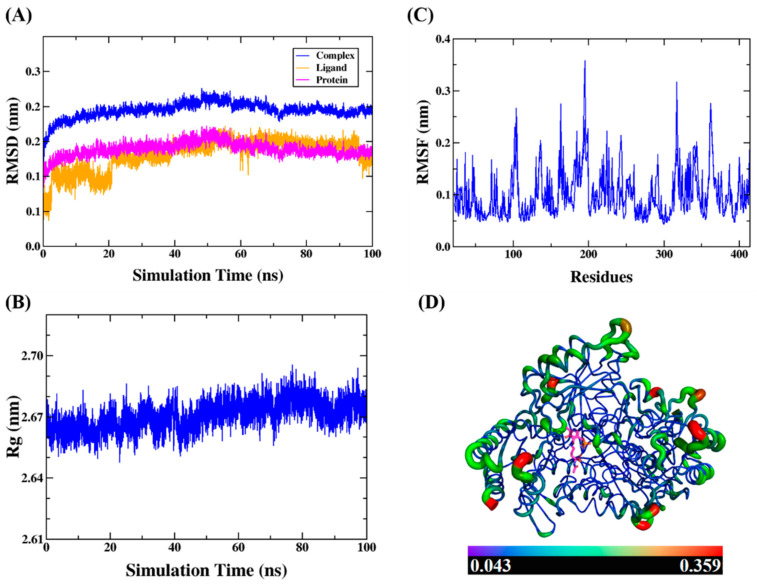
Structural stability and flexibility analysis of the *Cs*AcOAT–PLP-AcOrn complex during MD simulations. (**A**) RMSD profiles of the protein, ligand, and protein–ligand complex over the 100 ns simulation. (**B**) Rg profile of the *Cs*AcOAT–PLP-AcOrn complex during the 100 ns simulation. (**C**) RMSF profile of the *Cs*AcOAT–PLP-AcOrn complex, showing the residue-level flexibility throughout the simulation. (**D**) B-factor distribution mapped from RMSF values onto the three-dimensional structure of the *Cs*AcOAT–PLP-AcOrn complex. Regions with lower B-factor values represented by blue, whereas higher B-factor regions indicated by red. The ligand of PLP-AcOrn is shown in sticks with magenta carbons.

To evaluate whether the docked complex remained stable during simulation, we carried out a 100 ns MD run for the *Cs*AcOAT-PLP-AcOrn complex. Root mean square deviation (RMSD) and radius of gyration (Rg) are among the most commonly used metrics in MD trajectory analysis to evaluate conformational stability and structural compactness of biomolecular systems [45]. The RMSD profiles showed an initial adjustment phase followed by stabilization of both the protein backbone and the overall complex (Figure 8A). The ligand RMSD fluctuated more than the protein RMSD, as expected for a small molecule within a binding pocket, but did not show sustained drift indicative of dissociation. The radius of gyration (Rg) remained nearly constant throughout the trajectory (Figure 8B), indicating that the protein scaffold stayed compact.

To further assess local flexibility, root mean square fluctuation (RMSF) analysis was performed for the protein residues [45]. As shown in the RMSF profiles (Figure 8C), most residues exhibited relatively low fluctuation amplitudes, with values mainly distributed within 0.05–0.15 nm. This result indicates that the overall conformation of the complex remained stable throughout the simulation and that the structural framework of *Cs*AcOAT was well maintained after binding to PLP-AcOrn. Only a few localized regions showed relatively higher flexibility, with several distinct peaks reaching approximately 0.25–0.35 nm.

To visualize the spatial distribution of residue mobility, the RMSF values were further mapped onto the three-dimensional structure of *Cs*AcOAT–PLP-AcOrn complex in the form of a B-factor representation. Consistent with the RMSF analysis, the B-factor map showed that most regions of the protein were characterized by relatively low mobility, as indicated by the predominant blue-to-cyan coloring, forming a structurally stable core. In contrast, the higher B-factor signals were confined to several surface-exposed loop regions and terminal segments (Figure 8D). The absence of pronounced high-B-factor regions within the core and ligand-binding region further supports the structural integrity of the complex. Collectively, these results suggest that the *Cs*AcOAT–PLP-AcOrn complex adopts a stable overall conformation, with a rigid catalytic center and only limited flexibility in peripheral regions, which may be favorable for maintaining productive ligand binding and catalytic function.

**Figure 9:**
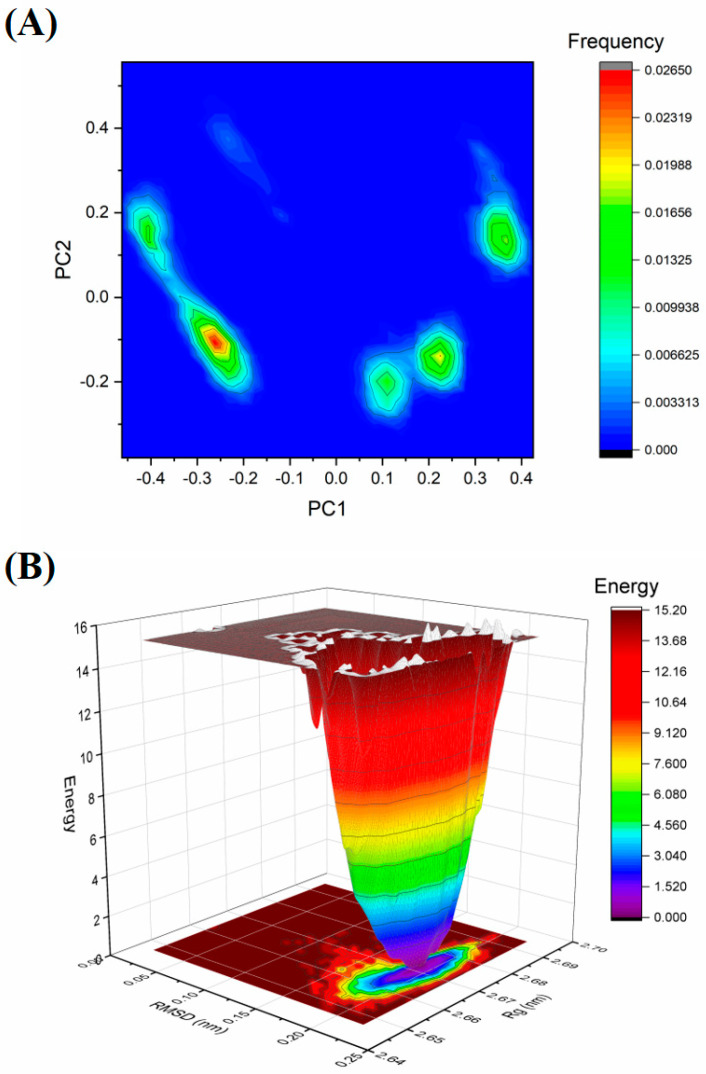
Conformational landscape analysis during MD simulations. (**A**) PCA of the PLP-AcOrn ligand trajectory projected onto the first two principle components (PC1 and PC2). (**B**) FEL of the *Cs*AcOAT–PLP-AcOrn complex constructed using the RMSD and Rg.

Furthermore, the ligand centroid distance and buried solvent-accessible surface area (Buried SASA) reached stable levels, indicating sustained contacts (Figure S6). Hydrogen-bond analysis provided additional evidence for persistent protein–ligand interactions. The total number of hydrogen bonds between the ligand and the protein fluctuated mainly between 6 and 10 during the simulation, indicating the presence of sustained polar contacts (Figure S6). Principal component analysis (PCA) of the ligand trajectory revealed a predominant high-frequency cluster together with several less populated conformational regions, indicating that the ligand mainly occupied one favored conformational space (Figure 9A). Consistently, the free energy landscape (FEL) generated from RMSD and Rg showed a major low-energy basin (Figure 9B), suggesting that the complex was primarily stabilized in a dominant conformational state. The agreement between PCA and FEL analyses supports a relatively stable conformational ensemble during the simulation.

### 3.7. Active Site Analysis Based on Docking Results

**Figure 10:**
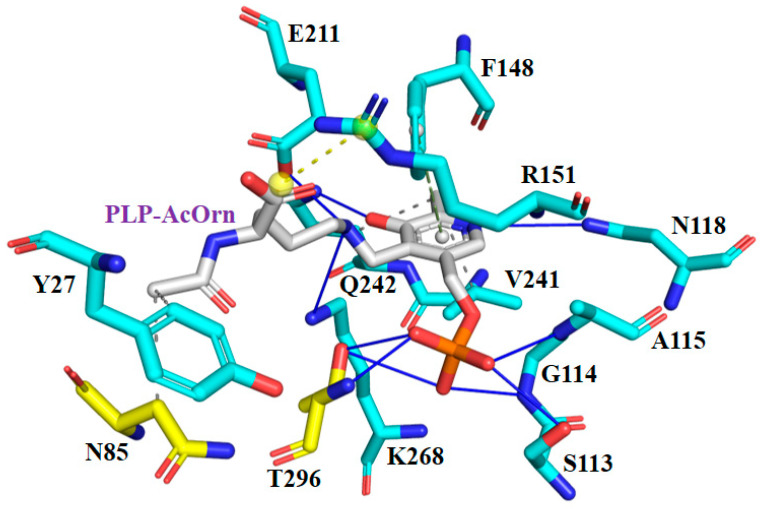
Predicted interactions between PLP-AcOrn and active-site residues of *Cs*AcOAT. Solid blue lines indicate hydrogen bonds, gray dashed lines indicate hydrophobic interactions, green dashed lines indicate π-stacking interactions, and yellow dashed lines indicate salt bridges. Yellow spheres represent charge centers, and gray spheres represent aromatic ring centers. Carbon atoms from chain A and chain B are colored in cyan and yellow, respectively. The carbon atom in PLP-AcOrn is colored in gray. The interaction is analyzed by PLIP.

Molecular docking and MD simulations indicated that PLP-AcOrn adopts a reliable and catalytically relevant binding pose in the active site of *Cs*AcOAT. The active site residues of *Cs*AcOAT and their interactions with PLP-AcOrn were examined in detail (Figure 10 and Figure S7). PLP-AcOrn was well accommodated within the catalytic pocket through a network of hydrogen bonds, hydrophobic contacts, salt bridges, and π-stacking interactions (Figure 10). Ser113, Gly114, Ala115, Asn118, Glu211, Gln242, Lys268, and Thr296 were predicted to form hydrogen bonds with PLP-AcOrn, while Tyr27, Asn85, Val241 and Gln242 contributed additional hydrophobic interactions (Figure 10). Phe148 and Arg151 were involved in π-stacking and salt bridge interactions, respectively (Figure 10). Notably, the side-chain amino group of Lys268 was positioned close to the imine bond of the PLP-AcOrn ketimine complex (Figure 10), consistent with the well-established role of the catalytic lysine in PLP-dependent aminotransferase [46,47,48]. The model of *Cs*AcOAT therefore fits well with the canonical AcAOTs. However, not all predicted contacts are necessarily equivalent in functional importance, and mutational analysis is required to distinguish essential catalytic residues from residues that mainly stabilize the local binding environment.

### 3.8. Site-Directed Mutagenesis and Functional Verification of Key Residues

To test the structural predictions, we measured the steady-state kinetic parameters of several active-site variants (Table 1). Substitution of Gly114, Asp239, Lys268 or Thr296 with alanine resulted in complete loss of catalytic activity toward both AcOrn and α-KG. This result strongly supports essential roles for these residues in catalysis or in maintaining a catalytically competent PLP-binding configuration.

In PLP-dependent aminotransferases, the conserved Asp residue typically stabilizes the protonated pyridine nitrogen of PLP through hydrogen bonding or electrostatic interaction (Figure S8), thereby enhancing the electron-sink function of the cofactor [49,50]. For instance, mutation of the corresponding Asp222 residue to Ala or Asn in *E. coli* aspartate aminotransferase reduced the catalytic rate to only 0.1% or 0.03% of the wild-type level, respectively [51]. The complete loss of activity in D239A is therefore consistent with a central role for Asp239 in maintaining the reactive PLP state.

The inactivity of K268A fits the canonical role of the catalytic lysine, which forms the internal Schiff base with PLP and initiate the transamination cycle [46]. Likewise, the T296A result suggests that Thr296 is critical for PLP stabilization, most likely through hydrogen bonding to the phosphate group of the cofactor (Figure 10). The complete loss of activity in G114A further indicates that Gly114 is not a passive backbone residue; rather, it appears to be required for the geometry of the catalytic pocket or the integrity of the local hydrogen-bonding network.

In contrast, S113A, A115T, and Q242A variants retained measurable activity, allowing a more detailed comparison. For these mutants, the *K_M_* values changed only modestly relative to wild-type *Cs*AcOAT, whereas the decreases in *k*_cat_ and *k*_cat_/*K_M_* were much more pronounced. This pattern suggests that Ser113, Ala115, and Gln242 are less important for initial substrate binding than for productive turnover after binding has occurred.

For S113A, the *k*_cat_ values decreased from 2.2 to 0.24 s^−1^ with AcOrn and from 2.0 to 0.22 s^−1^ with α-KG. The corresponding *k*_cat_/*K_M_* values dropped to 1.60 × 10^3^ and 8.15 × 10^3^ M^−1^·s^−1^, representing only about 12.4% and 8.2% of wild-type levels, respectively. A115T retained only 5.71 × 10^2^ and 2.50 × 10^3^ M^−1^·s^−1^ catalytic efficiency toward AcOrn and α-KG, respectively, corresponding to about 4.4% and 2.5% of the wild-type values, respectively. Q242A also showed substantially reduced turnover, with *k*_cat_/*K_M_* values of 1.67 × 10^−3^ and 5.71 × 10^−3^ M^−1^·s^−1^ toward AcOrn and α-KG, respectively, equivalent to about 12.9% and 5.7% of wild-type activity, respectively.

The S113T result was particularly informative. Although serine and threonine differ only by one methyl group, replacement of Ser113 with Thr sharply reduced catalytic performance. The *K_M_* values increased only moderately, to 0.35 mM for AcOrn and 0.036 mM for α-KG, but the *k*_cat_ values dropped sharply to 0.035 and 0.032 s^−1^, respectively. As a result, the catalytic efficiencies toward AcOrn and α-KG decreased to 1.00 × 10^2^ and 8.89 × 10^2^ M^−1^·s^−1^, respectively, corresponding to only about 0.8% and 0.9% of the wild-type levels, respectively. This disproportionate loss of turnover implies that the microenvironment around Ser113 is tightly constrained and sensitive to even a small increase in side-chain bulk. In other words, this position appears to contribute to catalytic geometry rather than simply to ligand capture.

Overall, the mutational data demonstrate that Asp239, Lys268, Thr296, and Gly114 are indispensable for activity, whereas Ser113, Ala115, and Gln242 contribute mainly to catalytic turnover and maintenance of a productive PLP-centered environment. The agreement between sequence conservation, structural modeling, docking, and mutagenesis strengthens this interpretation.

## 4. Conclusions

In this study, the AcOAT encoded by the *cce_3094* gene from *C. subtropica* ATCC 51142 (*Cs*AcOAT) was cloned, expressed, purified, and biochemically characterized. *Cs*AcOAT functions as a PLP-dependent aminotransferase acting on AcOrn and α-KG, with a clear preference for α-KG based on both substrate affinity and catalytic efficiency. The enzyme showed maximal activity at pH 8.5 and 30 °C, retained substantial activity over a broad temperature range, and responded differentially to metal ions, with Zn^2+^ and Co^2+^ acting as activators and Ni^2+^ as an inhibitor under the tested condition. Structural modeling, molecular docking, and MD simulations consistently supported a conserved AcOAT fold and a stable PLP-AcOrn binding mode in the catalytic pocket. Mutational analysis further identified Gly114, Asp239, Lys268, and Thr296 as essential residues for activity, whereas Ser113, Ala115, and Gln242 mainly affected catalytic turnover rather than substrate binding. These data support a conserved PLP-dependent catalytic framework in *Cs*AcOAT.

## Figures and Tables

**Table 1:** The kinetic parameters of *Cs*AcOAT and its variants.

Protein	Substrate	*K_M_* (mM)	*k*_cat_ (s^−1^)	*k*_cat_/*K_M_* (M^−1^·s^−1^)
wild-type	AcOrn	0.17 ± 0.03	2.2 ± 0.1	1.29 × 10^4^
	α-KG	0.020 ± 0.003	2.0 ± 0.2	1.00 × 10^5^
S113A	AcOrn	0.15 ± 0.02	0.24 ± 0.02	1.60 × 10^3^
	α-KG	0.027 ± 0.004	0.22 ± 0.01	8.15 × 10^3^
A115T	AcOrn	0.21 ± 0.08	0.12 ± 0.01	5.71 × 10^2^
	α-KG	0.06 ± 0.01	0.15 ± 0.02	2.50 × 10^3^
Q242A	AcOrn	0.09 ± 0.02	0.15 ± 0.02	1.67 × 10^3^
	α-KG	0.028 ± 0.007	0.16 ± 0.01	5.71 × 10^3^
S113T	AcOrn	0.35 ± 0.09	0.035 ± 0.002	1.00 × 10^2^
	α-KG	0.036 ± 0.01	0.032 ± 0.002	8.89 × 10^2^
K268A	AcOrn	NA	NA	NA
	α-KG	NA	NA	NA
D239A	AcOrn	NA	NA	NA
	α-KG	NA	NA	NA
T296A	AcOrn	NA	NA	NA
	α-KG	NA	NA	NA
G114A	AcOrn	NA	NA	NA
	α-KG	NA	NA	NA

NA: No Activity.

## Data Availability

The data presented in this study are available in the manuscript and in the Supplementary Materials.

## Supplementary Materials

The following supporting information can be downloaded at https://www.mdpi.com/article/10.3390/life16071212/s1, Table S1: Primer sequences used in this study; Table S2: Buffer systems of different pH values used in this study; Table S3: Amino acid sequence similarities among various AcOATs; Table S4: Cluster analysis of molecular docking conformations; Figure S1: The purification of *Cs*AcOAT wild-type protein; Figure S2: Evaluation of the *Cs*AcOAT structural model generated by SWISS-MODEL; Figure S3: Structural superposition of the three predicted models of *Cs*AcOAT; Figure S4: Evaluation of the *Cs*AcOAT structural model predicted by Alphafold; Figure S5: Structural superposition of the modeled *Cs*AcOAT structure with PLP-dependent aminotransferases; Figure S6: Interaction profiles of the protein–ligand complex during the 100 ns MD simulations; Figure S7: Interaction analysis of *Cs*AcOAT with PLP-AcOrn by PLIP; Figure S8: Predicted interactions between PLP and active-site residues of *Cs*AcOAT.
