# Supplementary material for: Biochemical Characterization and Active-Site Analysis of N-Acetylornithine Aminotransferase from Crocosphaera subtropica ATCC 51142
Source: Life (Basel). 2026 Jul 22;16(7):1212. doi: 10.3390/life16071212 (PMC13412850; doi:10.3390/life16071212)
Supplement: Supplementary file 1 [file life-16-01212-s001.zip › life-4445784-supplementary.pdf]

Biochemical Characterization and Active-Site Analysis of *N*-acetylornithine Aminotransferase from *Crocosphaera subtropica* ATCC 51142

Liyang Huang[1], Zhi-Min Li[2,*], Luna Gao[1], Siqi Wang[1], Zhifeng Wu[1], and Zhimin Li[1,*]

[1]College of Bioscience and Bioengineering, Jiangxi Engineering Laboratory for the Development and Utilization of Agricultural Microbial Resources, Jiangxi Agricultural University, Nanchang, Jiangxi 330045, China

[2]College of Chemistry and Materials, Jiangxi Agricultural University, Nanchang, Jiangxi 330045, China

* Correspondence: lzm_70@jxau.edu.cn (Z.-M.L.); zhiminli@jxau.edu.cn (Z.L.)

Tables

Table S1. Primer sequences used in this study

Table S2. Buffer systems of different pH values used in this study

Table S3. Amino acid sequence similarities among various AcOATs

Table S4. Cluster analysis of molecular docking conformations

Table S1. Primer sequences used in this study

Proteins	Primers	Primer Sequences (5'-3')
wild-type	*cce3094*-F	AACTTAT***CATATG***ACCCATACTACCCTTACAG
	cce3094-R	TCG***CTCGAG***CTAATTAACTGTAGCAGCACA
S113A	S113A-F	GTGTTTTTCTGTAAT***GCG***GGGGCAGAAG
	S113A-R	***CGC***ATTACAGAAAAACACCTTATCCGCACAAG
G114A	G114A-F	TTTTCTGTAATTCTG***C***GGCAGAAGCCA
	G114A-R	***G***CAGAATTACAGAAAAACACCTTATCCGCAC
Q242A	Q242A-F	GTATTCGATGAGGTA***GCG***GTAGGAGTTGGC
	Q242A-R	***CGC***TACCTCATCGAATACCAAGAGGATATTG
K268A	K268A-F	GTGTTAACCAGTGCG***GCG***GGGTTAGCCG
	K268A-R	***CGC***CGCACTGGTTAACACATCCGGTTCA
D239A	D239A-F	TCCTCTTGGTATTCG***CG***GAGGTACAAG
	D239A-R	***CG***CGAATACCAAGAGGATATTGTTTTCATCAC
T296A	T296A-F	TGGAACCCACGCTAGT***G***CCTTTGGAGG
	T296A-R	***C***ACTAGCGTGGGTTCCAGGGGTCAAAAC
S113T	S113T-F	GTGTTTTTCTGTAAT***ACC***GGGGCAGAAG
	S113T-R	***GGT***ATTACAGAAAAACACCTTATCCGCAC
A115T	A115T-F	TTCTGTAATTCTGGG***ACC***GAAGCCAAC
	A115T-R	***GGT***CCCAGAATTACAGAAAAACACCTTATC

Note: endonuclease sites are marked in purple italic bold. Mutation sites are marked in red italic bold.

Table S2. Buffer systems of different pH values used in this study

pH	buffers	Full Name	Concentration (mM)
6.0-6.5	Bis-Tris	Bis(2-hydroxyethyl)amino-tris(hydroxymethyl)methane	200
7.0-7.5	HEPES	*N*-2-hydroxyethylpiperazine-*N*-2-ethane sulfonic acid	200
8.0-8.5	Tris-HCl	Tris(hydroxymethyl)aminomethane hydrochloride	200
9.0	CHES	*N*-cyclohexyltaurine	200

Table S3. Amino acid sequence similarities among various AcOATs

Similarities (%)	*Cs*AcOAT	2ORD	8HT2	7NN1	3NX3	1WKH	5VIU
*Cs*AcOAT	100.00						
2ORD	47.14	100.00					
8HT2	40.11	47.25	100.00				
7NN1	38.97	41.35	55.79	100.00			
3NX3	37.66	43.12	35.60	32.62	100.00		
1WKH	36.83	43.28	41.42	44.01	36.77	100.00	
5VIU	35.38	33.25	37.07	35.60	30.43	33.85	100.00

Table S4. Cluster analysis of molecular docking conformations

Mode	PLP-AcOrn			PLP		
	Affinity (kcal/mol)	RMSD l.b.	RMSD u.b.	Affinity (kcal/mol)	RMSD l.b.	RMSD u.b.
1	-8.1	0.000	0.000	-7.9	0.000	0.000
2	-7.9	3.705	5.489	-6.8	2.192	3.146
3	-7.8	19.017	22.225	-6.8	19.663	21.583
4	-7.7	3.633	5.661	-6.7	18.288	20.794
5	-7.7	2.813	4.778	-6.6	17.529	19.818
6	-7.5	2.606	4.451	-6.5	3.276	5.434
7	-7.4	18.666	21.785	-6.4	19.129	21.364
8	-7.2	18.425	21.511	-6.3	18.425	21.105
9	-7.1	3.419	5.509	-6.2	20.260	22.447
10	-7.0	21.060	23.816	-6.1	15.978	18.233
11	-6.8	24.645	27.631	-6.0	19.207	21.672
12	-6.8	22.937	25.895	-6.0	3.105	5.840
13	-6.7	2.882	4.928	-5.9	3.405	4.436
14	-6.7	18.274	21.502	-5.9	22.670	24.159
15	-6.6	26.700	28.910	-5.6	22.852	24.680
16	-6.6	2.858	4.831	-5.6	22.743	24.916
17	-6.4	23.807	26.832	-5.5	23.613	25.453
18	-6.2	24.562	28.138	-5.5	22.845	25.102
19	-6.2	25.470	28.123	-5.3	22.753	24.301
20	-6.1	25.249	28.462	-5.2	23.298	25.476

Figure Legends

Figure S1. The purification of *Cs*AcOAT wild type protein. M: mixed broad molecular weight protein standards; 1: Cell lysate after induction of expression; 2: Supernatant after cell disruption and centrifugation; 3: Resuspended cell pellet; 4: Supernatant flow through Ni-NTA flow through; Lane 5~13: 20-200 mmol/L imidazole elution.

Figure S2. Evaluation of the *Cs*AcOAT structural model generated by SWISS-MODEL. Model quality assessment yielded a GMQE score of 0.76, a global QMEANDisCo score of 0.78, and local QMEANDisCo scores above 0.6 for more than 90% of the residues.

Figure S3. Structural superposition of the three predicted models of *Cs*AcOAT. The models generated by SWISS-MODEL, AlphaFold and RoseTTAFold are shown in cyan, grey and green, respectively. The RMSD values between the SWISS-MODEL and AlphaFold structures, the SWISS-MODEL and RoseTTAFold structures, and the AlphaFold and RoseTTAFold structures are 0.677 Å, 0.907 Å, and 0.759 Å, respectively.

Figure S4. Evaluation of the *Cs*AcOAT structural model predicted by Alphafold. The model yielded a pTM score of 0.95, with very high pLDDT values across most regions.

Figure S5. Structural superposition of the modeled *Cs*AcOAT structure with PLP-dependent aminotransferases. The structure of *Cs*AcOAT was generated using Swiss-Model and is shown in cyan. The crystal structure of gamma-aminobutyrate aminotransferase from Corynebacterium glutamicum (PDB ID: 6J2V) is shown in magenta, while that of aspartate aminotransferase from Thermus thermophilus HB8 (PDB ID: 1GCK) is shown in yellow. The RMSD values between CsAcOAT and 6J2V and between CsAcOAT and 1GCK are 1.127 Å and 1.837 Å, respectively.

Figure S6. Interaction profiles of the protein–ligand complex during the 100 ns MD simulations. (**A**) Time evolution of the minimum distances between the ligand and the binding pocket, and between the ligand and the protein. (**B**) Buried solvent-accessible surface area (buried SASA) of the ligand within the binding site over the simulation time. (**C**) Number of hydrogen bonds formed between the protein and ligand throughout the simulation.

Figure S7. Interaction analysis of *Cs*AcOAT with PLP-AcOrn by PLIP.

Figure S8. Predicted interactions between PLP and active-site residues of *Cs*AcOAT. Solid blue lines indicate hydrogen bonds, gray dashed lines indicate hydrophobic interactions, and green dashed lines indicate π-stacking interactions. Gray spheres represent aromatic ring centers. Carbon atoms from chain A and chain B are colored in cyan and yellow, respectively. The carbon atom in PLP is colored in gray. The interaction is analyzed by PLIP.

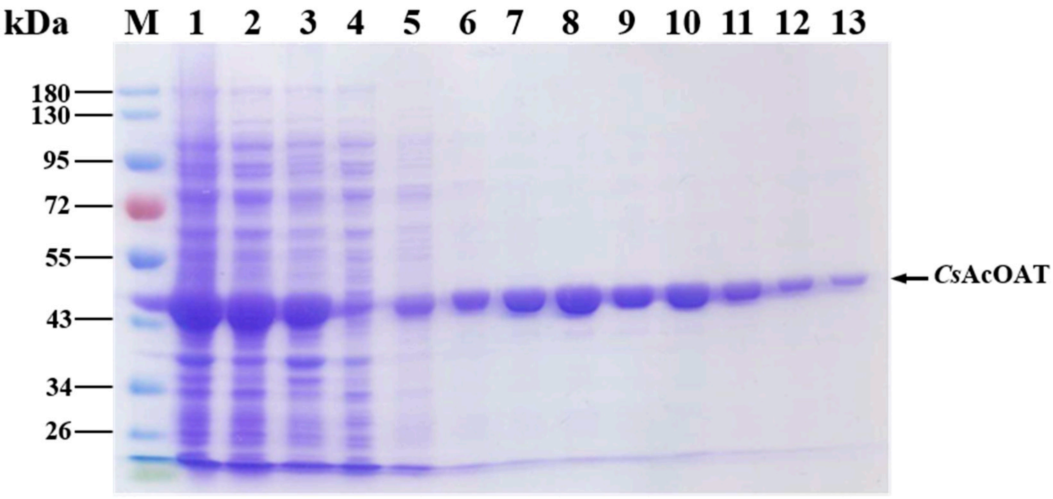

Figure S1. The purification of *Cs*AcOAT wild type protein. M: mixed broad molecular weight protein standards; 1: Cell lysate after induction of expression; 2: Supernatant after cell disruption and centrifugation; 3: Resuspended cell pellet; 4: Supernatant flow through Ni-NTA flow through; Lane 5~13: 20-200 mmol/L imidazole elution.

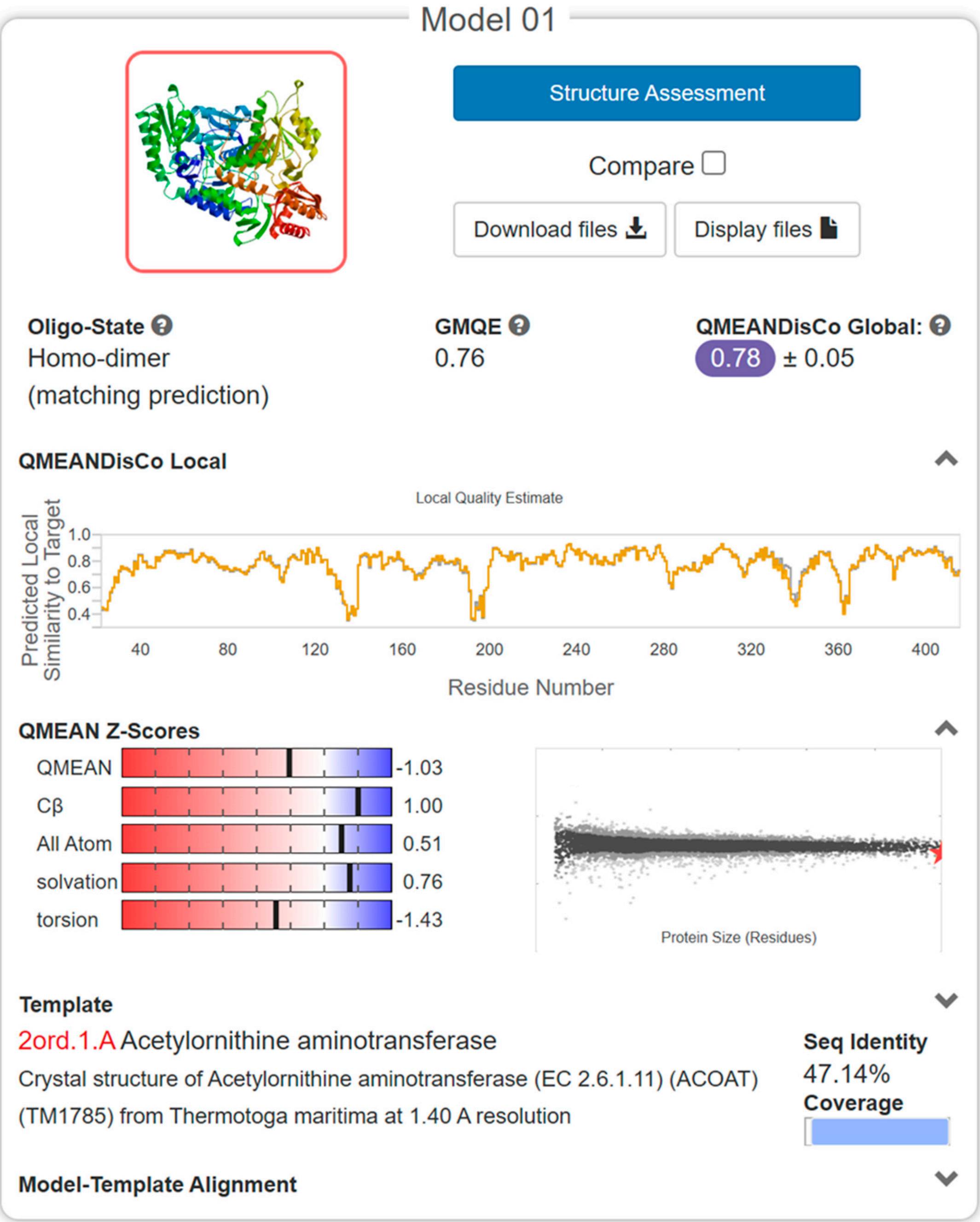

Figure S2. Evaluation of the *Cs*AcOAT structural model generated by SWISS-MODEL. Model quality assessment yielded a GMQE score of 0.76, a global QMEANDisCo score of 0.78, and local QMEANDisCo scores above 0.6 for more than 90% of the residues.

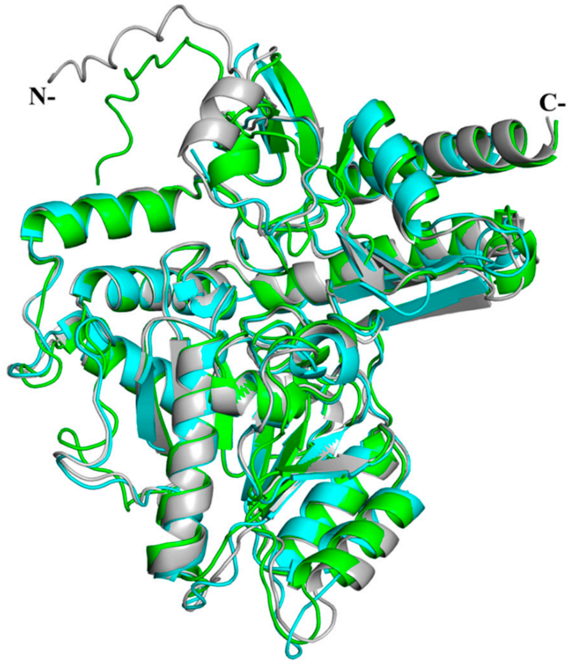

Figure S3. Structural superposition of the three predicted models of *Cs*AcOAT. The models generated by SWISS-MODEL, AlphaFold and RoseTTAFold are shown in cyan, grey and green, respectively. The RMSD values between the SWISS-MODEL and AlphaFold structures, the SWISS-MODEL and RoseTTAFold structures, and the AlphaFold and RoseTTAFold structures are 0.677 Å, 0.907 Å, and 0.759 Å, respectively.

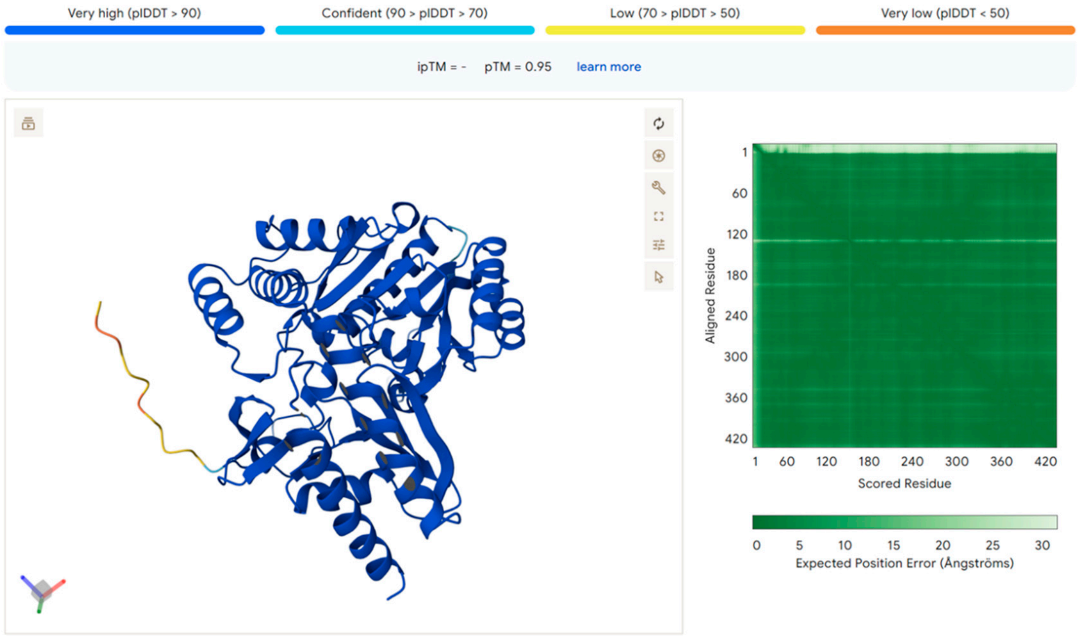

Figure S4. Evaluation of the *Cs*AcOAT structural model predicted by Alphafold. The model yielded a pTM score of 0.95, with very high pLDDT values across most regions.

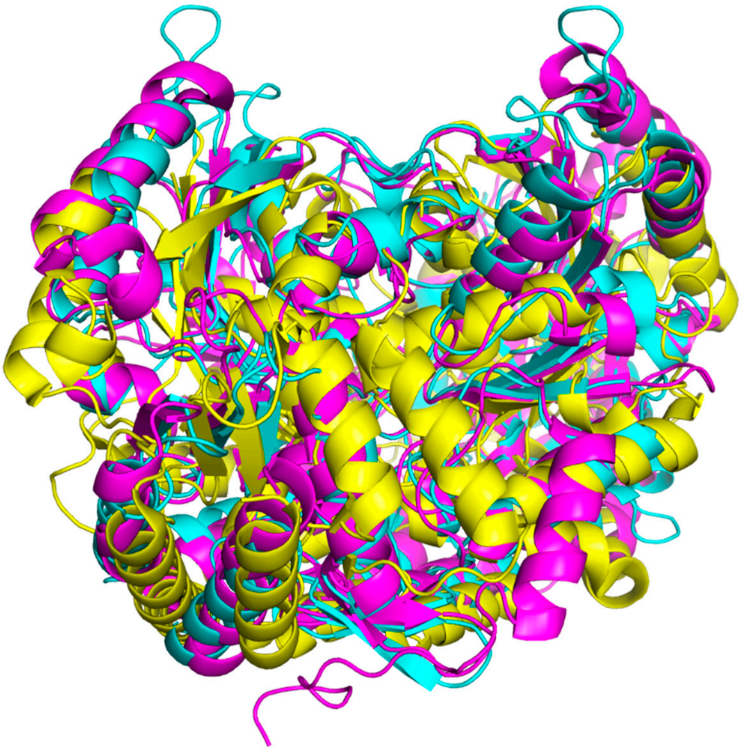

Figure S5. Structural superposition of the modeled *Cs*AcOAT structure with PLP-dependent aminotransferases. The structure of *Cs*AcOAT was generated using Swiss-Model and is shown in cyan. The crystal structure of gamma-aminobutyrate aminotransferase from *Corynebacterium glutamicum* (PDB ID: 6J2V) is shown in magenta, while that of aspartate aminotransferase from *Thermus thermophilus* HB8 (PDB ID: 1GCK) is shown in yellow. The RMSD values between *Cs*AcOAT and 6J2V and between *Cs*AcOAT and 1GCK are 1.127 Å and 1.837 Å, respectively.

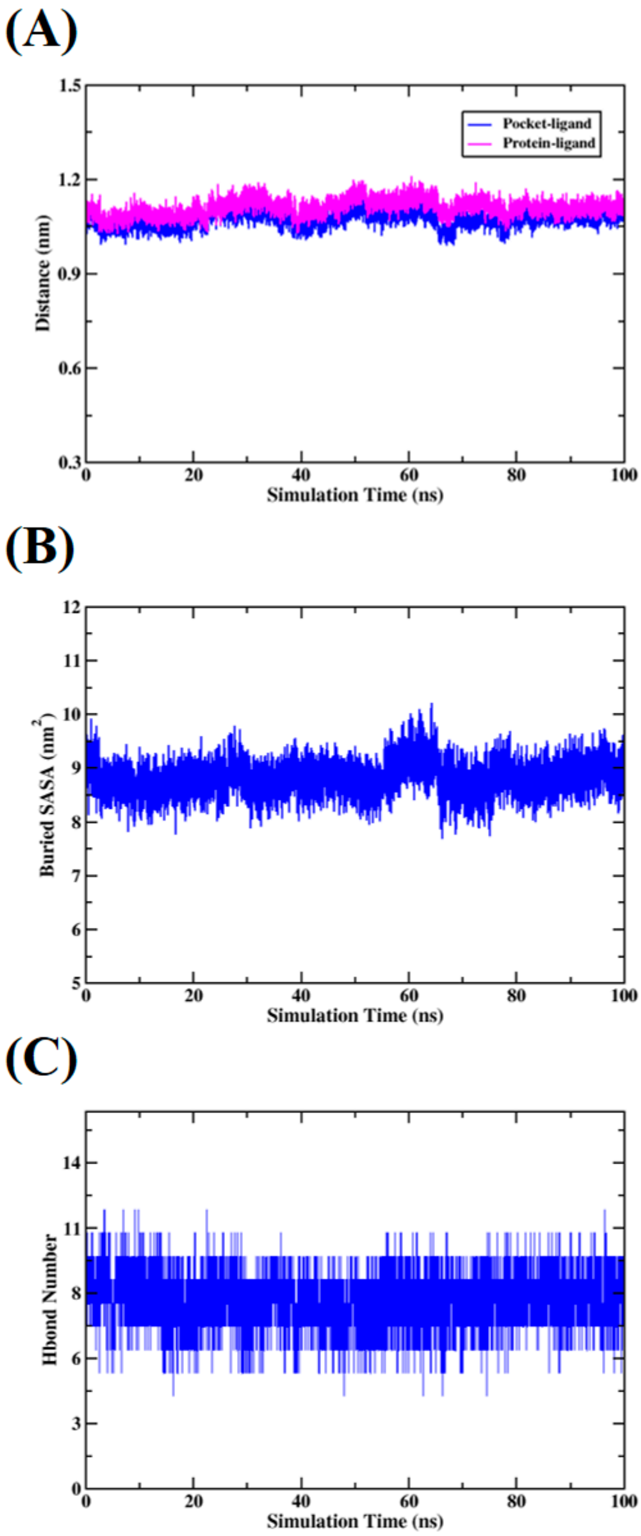

Figure S6. Interaction profiles of the protein–ligand complex during the 100 ns MD simulations. (**A**) Time evolution of the minimum distances between the ligand and the binding pocket, and between the ligand and the protein. (**B**) Buried solvent-accessible surface area (buried SASA) of the ligand within the binding site over the simulation time. (**C**) Number of hydrogen bonds formed between the protein and ligand throughout the simulation.

Hydrophobic Interactions

Index	Residue	AA	Distance	Ligand Atom	Protein Atom
1	27A	TYR	3.76	9	84
2	85B	ASN	3.88	9	3572
3	241A	VAL	3.84	6	1772
4	242A	GLN	3.93	6	1779

Hydrogen Bonds

Index	Residue	AA	Distance H-A	Distance D-A	Donor Angle	Protein donor?	Side chain	Donor Atom	Acceptor Atom
1	113A	SER	2.99	3.30	100.47	✓	✓	766 [O3]	31 [O2]
2	114A	GLY	2.20	3.15	160.34	✓	×	767 [Nam]	27 [O3]
3	115A	ALA	2.35	3.30	161.95	✓	×	771 [Nam]	31 [O2]
4	118A	ASN	3.51	4.05	116.76	✓	✓	796 [Nam]	21 [Nar]
5	211A	GLU	2.70	3.25	114.51	×	✓	7 [N3]	1528 [O.co2]
6	242A	GLN	1.99	2.80	137.29	✓	✓	1782 [Nam]	29 [O3]
7	268A	LYS	3.02	3.91	145.19	✓	✓	1975 [N3+]	7 [N3]
8	296B	THR	2.78	3.63	146.62	×	✓	27 [O3]	5206 [O3]
9	296B	THR	2.06	2.96	152.00	✓	×	5200 [Nam]	26 [O3]
10	296B	THR	2.12	3.04	156.55	✓	✓	5206 [O3]	26 [O3]

π-Stacking

Index	Residue	AA	Distance	Angle	Offset	Stacking Type	Ligand Atoms
1	148A	PHE	5.01	87.16	1.57	T	1, 3, 5, 8, 10, 21

Salt Bridges

Index	Residue	AA	Distance	Protein positive?	Ligand Group	Ligand Atoms
1	151A	ARG	3.85	✓	Carboxylate	32, 33

Figure S7. Interaction analysis of *Cs*AcOAT with PLP-AcOrn by PLIP.

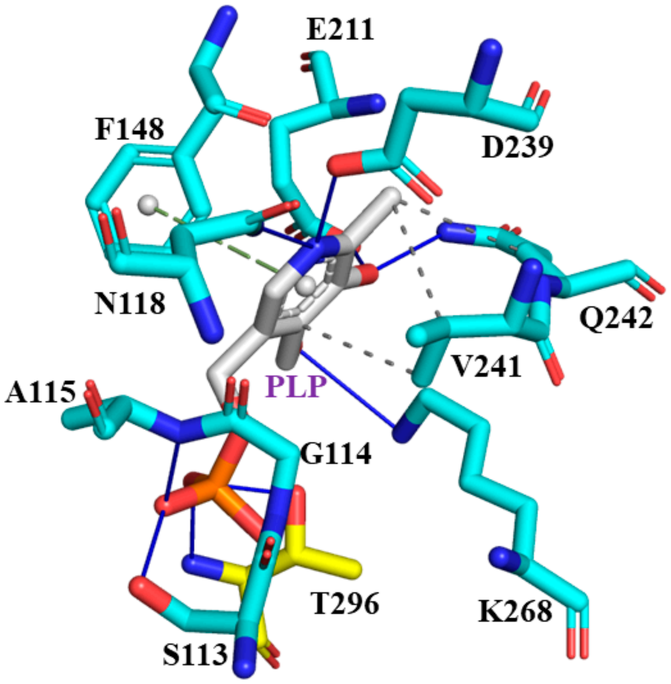

Figure S8. Predicted interactions between PLP and active-site residues of *Cs*AcOAT. Solid blue lines indicate hydrogen bonds, gray dashed lines indicate hydrophobic interactions, and green dashed lines indicate π-stacking interactions. Gray spheres represent aromatic ring centers. Carbon atoms from chain A and chain B are colored in cyan and yellow, respectively. The carbon atom in PLP is colored in gray. The interaction is analyzed by PLIP.
